# Dispersive solid phase microextraction based on magnesium oxide nanoparticles for preconcentration of auramine O and methylene blue from water samples

**DOI:** 10.1038/s41598-022-16948-z

**Published:** 2022-07-27

**Authors:** Weidong Li, Jianping Qiu, Leila Baharinikoo, T. CH. Anil Kumar, Basim Al-qargholi, Shafik S. Shafik, Reathab Abbass, Shelesh krishna Saraswat

**Affiliations:** 1 Hangzhou Normal University Qianjiang College, Hangzhou, 310018 China; 2 Zhejiang Normal University Xingzhi College, Jinhua, 321004 China; 3 Department of Analytical Chemistry, Faculty of Chemistry, University of Mazandaran, Babolsar, Iran; 4 Department of Mechanical Engineering, Vignan’s Foundation for Science Technology and Research, Vadlamudi, Guntur, India; 5 Biomedical Engineering Department, Al-Mustaqbal University College, 51001 Hillah, Babylon Iraq; 6 Experimental Nuclear Radiation Group, Scientific Research Center, Al-Ayen University, Nasiriyah, Thi-Qar Iraq; 7 Medical Technical College, Al-Farahidi University, Baghdad, Iraq; 8 Deprtment of Electronics and Communication, GLA University, Mathura, India

**Keywords:** Environmental sciences, Chemistry

## Abstract

In this study, we investigated the process of preconcentrate and determine trace amounts of Auramine O (AO) and methylene blue (MB) dyes in environmental water samples. For this purpose, the ultrasound-assisted dispersive-magnetic nanocomposites-solid-phase microextraction (UA-DMNSPME) method was performed to extract AO and MB from aqueous samples by applying magnesium oxide nanoparticles (MgO-NPs). The proposed technique is low-cost, facile, fast, and compatible with many existing instrumental methods. Parameters affecting the extraction of AO and MB were optimized using response surface methodology (RSM). Short extraction time, low experimental tests, low consumption of organic solvent, low limits of detection (LOD), and high preconcentration factor (PF) was the advantages of method. The PF was 44.5, and LOD for AO and MB was 0.33 ng mL^−1^ and 1.66 ng mL^−1^, respectively. The linear range of this method for AO and MB were 1–1000 ng mL^−1^ and 5–2000 ng mL^−1^, respectively. In addition, the relative standard deviation (RSD; n = 5) of the mentioned analytes was between 2.9% and 3.1%. The adsorption–desorption studies showed that the efficiency of adsorbent extraction had not declined significantly up to 6 recycling runs, and the adsorbent could be used several times. The interference studies revealed that the presence of different ions did not interfere substantially with the extraction and determination of AO and MB. Therefore, UA-DMNSPME-UV/Vis method can be proposed as an efficient method for preconcentration and extraction of AO and MB from water and wastewater samples.

## Introduction

Wastewater from various industries such as dyeing, wood, leather, and fish farming contain dyes and is considered the source of environmental pollution. Even low concentrations of these dyes can change the color of the water. Dyes are non-degradable and stable pollutants released into the environment in the same way along with untreated effluents of various industries^1,2^.

Auramine O (AO) and methylene blue (MB) are two dyes studied in the present research. AO contains solid yellow crystals, and MB has blue crystals. Also, AO and MB dyes are among the most widely used and important dyes for dyeing cotton, paper, wool, and silk. However, prolonged exposure to these dyes can cause localized burns, nausea, increased sweating, mental disorders, and even cancer in humans and animals^3–5^.

The sample preparation is the main step in an analysis process that guarantees to obtain the desired results. Sample preparation involves converting the real sample matrix into a state that is suitable for analysis by separation techniques or other methods. The extraction process is the most common sample preparation method^6^. This technique isolates and pre-concentrates trace amounts of analytes from the sample matrix.

Solid-phase microextraction (SPME) is a widely used technique for separating and preconcentrating organic and inorganic analytes from aqueous samples^7,8^. In this method, the desired species are adsorbed and concentrated using a solid phase or a solid coating. Next, the adsorbed species are washed with a proper solvent, followed by analysis and measurement by analytical instruments. Very low consumption of organic solvent, high recovery, low cost, and short extraction time are the major advantages of the SPME method^9,10^.

Several techniques exist for determining dyes present in different samples, e.g., high-performance liquid chromatography-ultraviolet (HPLC–UV), electrochemistry, and spectrophotometry^11–16^. Good selectivity, easy operation, low operating costs, and the ability to determine a wide range of materials in various fields are the main advantages of the spectrophotometry method over the other methods^17,18^.

Different adsorbents have been used in the SPME method, with nanoparticles being the most widely used ones. The advantages of extraction with nanoparticles are cost-effectiveness, eco-friendliness, low consumption of adsorbent, and high extraction percentage^19,20^.

In the present study, we used magnesium oxide nanoparticles (MgO-NPs) to extract dyes. Microcrystalline size, high adsorption capacity, ease and low cost of production, high surface area, and the presence of active sites are the beneficial properties of MgO-NPs. Regarding these chemical properties, MgO-NPs are widely used in water and wastewater treatment^21,22^.

Tian et al. (2020) used SPME for extracting phthalate esters (PAEs) from an aqueous solution using magnesium/aluminum-layered double hydroxide (Mg/Al-LDH). Under the optimized conditions, the linear ranges (LDs), limits of detection (LODs), and limits of quantification (LOQs) of the developed method were 1–500 μg L^−1^, 0.42–1.29 μg L^−1^, and 1.40–4.13 μg L^−1^, respectively. SPME method was successfully applied to analyze PAEs from real samples, and acceptable results were obtained^23^.

In another study, Tan et al. (2017) applied MgO microspheres functionalized with phenyl trichlorosilane (PTS-MgO) as an adsorbent to extract seven dioxin-like polycyclic aromatic hydrocarbons (DL-PAHs) via solid-phase dispersion (MSPD) extraction. Under the optimized conditions, the MSPD method combined with HPLC-FLD exhibited RSD < 9.6% and LODs of 0.02–0.12 ng g^−1^. Also, LOQs and LD were obtained in the range of 0.07–0.40 ng g^−1^ and 0.5–160 ng g^−1^, respectively^24^.

Wang et al. (2017) used the SPME method to preconcentrate and determine arsenic. First, magnesium oxide (MgO) was synthesized and used as a highly potential adsorbent for arsenic extraction with the ability to use sequentially. In their study, 1.5 mg MgO, pH 5.0, and 15 min ultrasonic time were selected as optimum reaction conditions. Under the optimal conditions, RSD (n = 7), LOD, and enrichment factor (EF) were about 4.5%, 0.087 μg L^−1^, and 13, respectively^25^.

Several factors with different levels can be considered in analytical and experimental studies. Therefore, if several factors have multiple levels in investigating a reaction, optimization with this vast number of experiments is not economically viable (i.e., the full factorial of the variables). As a result, instead of using full factorial experiments, the idea of utilizing partial factorial and experimental design has been proposed^26,27^. Nowadays, response surface methodology (RSM) is one of the simplest, fastest, and most feasible design methods used in many industries. Experimental design steps include selecting a suitable design according to the number of factors and their levels, conducting the experiments according to the design of experiments, and finally analyzing the results^28–30^.

Modern techniques such as ultrasound-assisted (UA) technology are proven methods to increase extraction performance compared to enzymatic and soxhlet extraction methods^31,32^. The main mechanism of ultrasonic-assisted extraction is attributed to the phenomenon called cavitation. Irradiating in ultrasound leads to the formation of micro-bubbles. Then, these micro-bubbles grow and reach their maximum point so that they cannot maintain their shape. Therefore, they collapse and cause high temperature and pressure (a phenomenon referred to as cavitation). In this phenomenon, molecules are temporarily detached from their sites and transfer as a sound wave that can collide with the surrounding molecules. When these bubbles collapse onto the solid surface, the high pressure and temperature released produce microjets and shock waves directed to the solid surface. These microjets impact the surface, leading to its wear, breakage, and degradation^33,34^.

This study investigates the extraction of AO and MB dyes via ultrasound-assisted dispersive-magnetic nanocomposites-solid phase microextraction (UA-DMNSPME), followed by determining the dyes by UV/Vis spectrophotometry (UA-DMNSPME-UV/Vis). Eventually, RSM is used to optimize the factors.

## Experimental

### Materials and instrumentation

All materials used in this study, including magnesium chloride hexahydrate (MgCl_2_.6H_2_O), sodium hydroxide (NaOH), hydrogen chloride (HCl), auramine O (C_17_H_22_ClN_3_), and methylene blue (C_16_H_18_ClN_3_S), were of analytical purity. These materials were purchased from Merck and Sigma-Aldrich companies. Solutions of AO (100 µg mL^−1^) and MB (100 µg mL^−1^) were prepared separately by dissolving their solid powder in an aqueous solution. NaOH and HCl (0.1 M) solutions were used to adjust the pH, and a pH meter (model: Metrohm 780) was applied to measure the pH. A UV/Vis spectrophotometer (model: Jasco V-670) was used to determine the dye concentrations, and an ultrasonic bath (model: Fisherbrand™ 11,203) was used to accelerate the separation phase of the extraction phase. Scanning electron microscopy (SEM) (model: KYKY-EM3200), energy-dispersive X-ray spectroscopy (EDX) (model: Link ISIS-300), X-ray diffractometer (XRD) (model: Philips PW 1800), and Brunauer–Emmett–Teller (BET) (model: Quantachrome NOVA 2200e) specific surface area analysis were employed to characterize the adsorbent structure. Finally, analyses were performed using the Design-Expert software version 10. The characteristics of dyes are listed in Table 1.

**Table 1:** Characteristics of the dyes.

Characteristic	Auramine O (AO)	Methylene blue (MB)
Molecular formula	C17H22ClN3	C16H18ClN3S
Molecular weight	303.83 (g mol ^−1^)	319.85 (g mol ^−1^)
Maximum Wavelength	430 nm	665 nm
Chemical structure		

### Synthesis of the magnesium oxide nanoparticles (MgO-NPs)

MgO nanoparticles were synthesized by the sol–gel method by dissolving 100 g MgCl_2_.6H_2_O in 500 mL distilled water in a beaker (1 L). Then, 50 mL NaOH solution (1 N) was added to the beaker. The mixture was stirred for 4 h (at 400 rpm) and temperature of 70 °C to form Mg(OH)_2_. Mg(OH)_2_ was also centrifuged at 4500 rpm for 5 min. The final precipitate was washed several times with distilled water and dried at 100℃ for 12 h. Finally, the dried powder was calcined at 400℃ for 3 h in an electrical furnace. The synthesized sample’s morphology, characterization, and size were evaluated using XRD, SEM, EDX, and BET analyses.

### Response surface methodology (RSM)

RSM is very efficient and cost-effective for experiments in which a response or a set of responses is affected by various parameters. This method optimizes the response influenced by several factors to obtain a mathematical relationship between variables and the response. RSM allows estimating the linear, second-order, and interaction effects and predicting a suitable model. Central composite design (CCD) is one of the most widely used methods in RSM. This design mostly requires five levels. When each experiment is assigned to a point, the design consists of three points: (1) the axial points, (2) the factorial points, and (3) the center points^35^. The number of experiments to perform in CCD is determined by Eq. (1).

1 $${\text{N}} = 2^{k} + \, 2K + \, C_{0}$$

where N is the number of parameters, 2^ k^ is the number of factorial experiments, 2 K is the number of axial experiments, and *C*_*0*_ is the number of central experiments. Factorial experiments are used to estimate the linearity of the model and the interaction between the model parameters. Moreover, axial experiments are performed to determine the upper and lower limits to obtain the degree of model curvature. Central experiments are done to estimate net error. The system behavior is described by a second-order polynomial equation (Eq. 2).

2 $${\text{Y}} = \beta_{0} + \mathop \sum \limits_{i = 1}^{k} \beta_{i} X_{i} + \mathop \sum \limits_{i = 1}^{k} \beta_{ii} X_{i}^{2} + \mathop \sum \limits_{i \le j}^{k} \mathop \sum \limits_{j}^{k} \beta ijX_{i} X_{j} + e$$

where Y is the extraction percentage or yield, *k* is the number of parameters, *β*_*0*_ is a constant, *β*_*i*_ is the coefficients of linear parameters, *β*_*ii*_ is the squared effect, *β*_*ij*_ and *β*_*ii*_ are the coefficients of the interacting parameters, *X*_*i*_ and *X*_*j*_ represent the variable, and *e* is the random error of experiments representing the difference or uncertainty between the predicted and measured values^36^.

### Recommended procedure

AO and MB extraction experiments were conducted by UA-DMNSPME-UV/Vis method. To this end, 10 mL of a solution containing AO (500 ng mL^−1^) and MB (500 ng mL^−1^) was transferred to a glass tube (15 mL). Then, 0.025 g of MgO-NPs as an adsorbent was added to this solution. The pH of the solution was adjusted to 7. The analyte adsorption on the adsorbent and its mass transfer was facilitated by placing the glass tube in an ultrasonic bath for 5 min. Afterward, the sample was centrifuged for 5 min (3500 rpm) to separate the phases well. The adsorbent was removed immediately by applying an external magnet, and the solution was decanted. The adsorbent was washed with 225 μL acetone, and 100 μL of the solvent containing the sample was drawn to a Hamilton syringe and placed in a microcell. At the end of adsorption, the analyte was determined with UV/Vis spectrophotometer at the maximum dye wavelength. All experiments were performed at 25℃. The extraction recovery was calculated by Eq. (3). According to this equation, the extraction recovery is defined as the percentage of the number of moles of analyte extracted into the acceptor phase (*n*_*f*_) divided by the number of moles of analyte initially presented in the sample solution (*n*_*a*_).

3 $${\text{ER}} = \frac{{n_{f} }}{{n_{a} }} \times {1}00 = {\text{ PF }} \times \frac{{V_{f} }}{{V_{a} }} \times 100$$

In this equation, *V*_a_ and *V*_f_ are the volumes of sample solution and acceptor phase, respectively. Equation (4) was applied to calculate the PF.

4 $${\text{PF}} = \frac{{C_{f} }}{{C_{a} }}$$

Based on this equation, PF is defined as the ratio of the concentration of analytes extracted into the extraction phase (*C*_*f*_) to the concentration of the analyte in the original aqueous sample (*C*_*a*_).

### Reusability studies

Since the used adsorbent is synthetic and made from laboratory materials, its regeneration and reusability are among the most important features in evaluating its performance. The experiments were conducted based on the procedure described in Section Recommended procedure to measure the adsorbent capacity. for this purpose, the adsorbent, which was used once to extract AO and MB dyes under optimum conditions, was separated from the solution by external magnet and was washed several times with acetone and distilled water. The adsorbent was again centrifuged and dried in an oven at 80℃ for 10 h to be reused in extraction experiments. This procedure was done for eight consecutive cycles.

## Results and discussion

### Characterization of sorbent (magnesium oxide nanoparticles (MgO-NPs))

**Figure 1:**
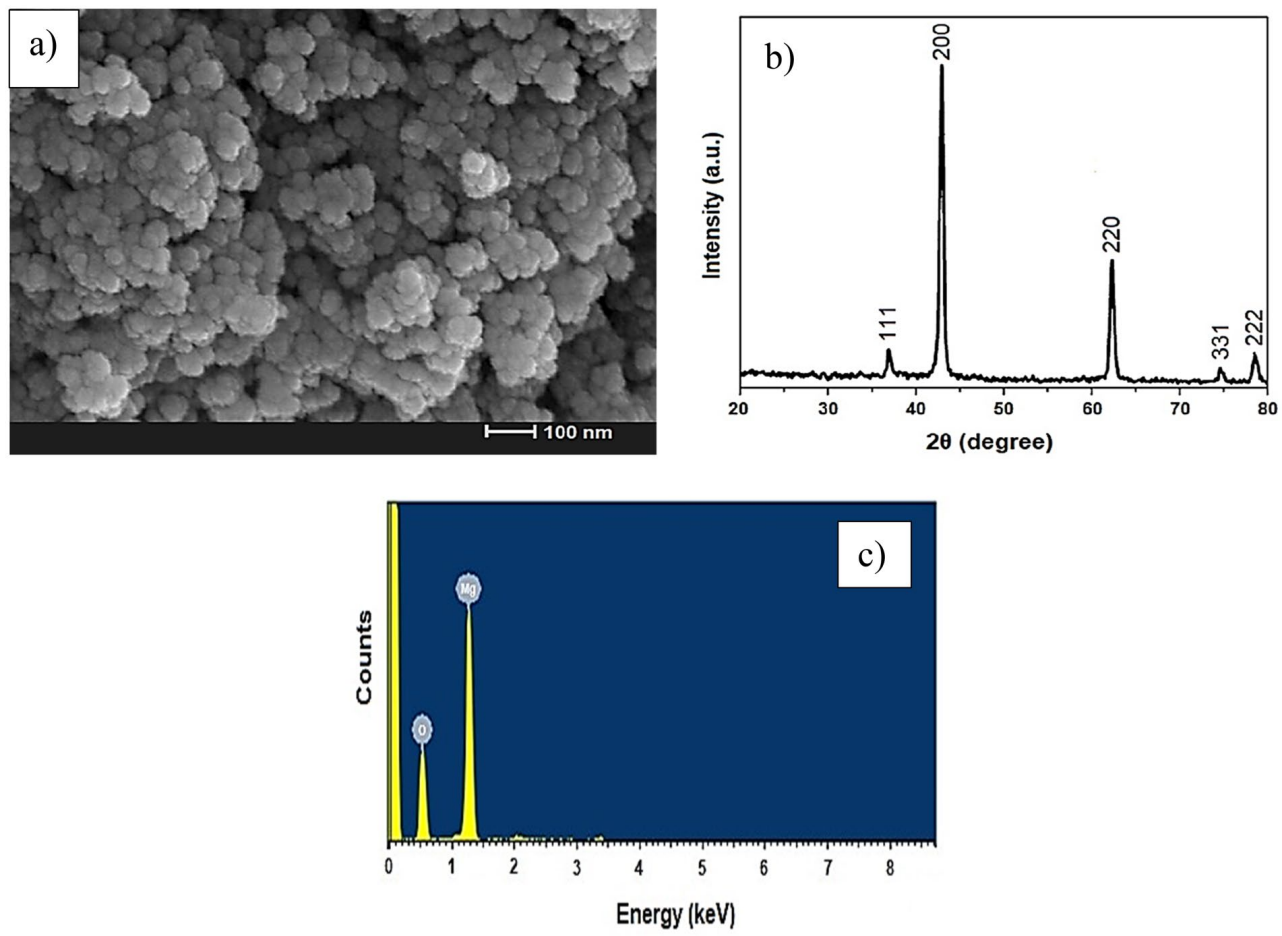
(**a**) SEM image, (**b**) X-ray diffraction pattern, and (**c**) EDX spectrum of MgO.

SEM was used to evaluate the surface morphology of the synthesized particles. As shown in Fig. 1a, the synthesized MgO-NPs are spheral and uniform with a good size distribution. The average particle size is 51.94 nm. The XRD pattern of MgO-NPs is shown in Fig. 1b. In this diffraction pattern, no impurity peaks are observed. The crystal structure of magnesium oxide nanoparticles is face-centered cubic due to the correspondence of its peaks with the standard card JCPDS no. 87–0653. The average particle size of the sample is 40.53 nm, according to the Debye Scherrer formula.

Figure 1c represents the energy-dispersive X-ray (EDX) of the MgO adsorbent. According to this figure, the presence of Mg and O peaks in the elemental analysis of the MgO adsorbent shows that the expected MgO is successfully formed, and no other elements are observed, representing the purity of the adsorbent surfaces.

Nitrogen adsorption–desorption measurements provide the available surface of MgO-NPs. Accordingly, BET surface area, pore volume, and pore size were 39.42 m^2^g^−1^, 40.37 cm^3^g^−1^, and 38.56 nm, respectively.

### Effects of type of extraction solvent

**Figure 2:**
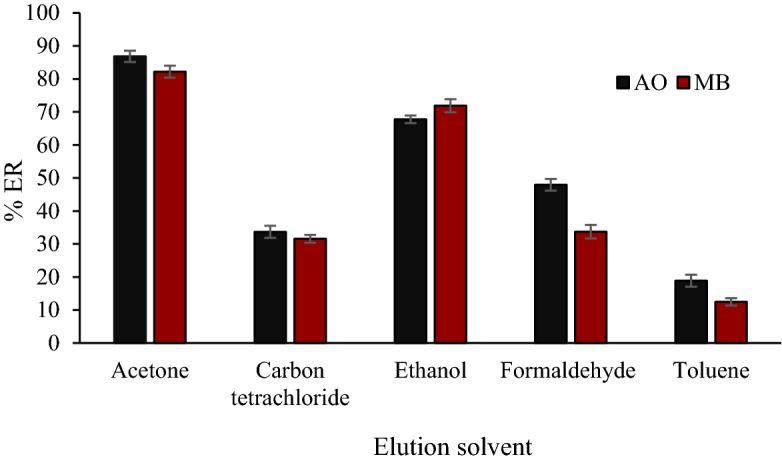
The effect of organic phase composition on the extraction efficiency (Extraction conditions: pH = natural, dye concentration 500 ng mL^−1^, adsorbent mass 0.03 g, and ultrasound time 5 min).

In this study, we also investigated the effect of solvent type on AO and MB extraction. For this purpose, toluene, ethanol, formaldehyde, acetone, and carbon tetrachloride were used. The extraction rate for AO and MB was measured with different solvents. The results (Fig. 2) show that solvent type significantly affects the extraction rate of AO and MB. The highest extraction rate of both analytes was obtained by acetone, followed by ethanol.

### Significant variable optimization by RSM

In this research, initial studies and experiments reveal four variables affecting the extraction process (adsorbent mass, sonication time, pH of solution, and eluent volume). After determining the effective range of these parameters, the CCD-based RSM method with four factors at five levels and six central points was used to design and optimize the multivariate preconcentration experiments. These variables were entered in the Design-Expert software. The software results indicate 30 experiments, including 30 runs, 16 factorial points, 6 central points, and 8 axial points. Axial points are points that add a constant value to the upper limit of the parameter and subtract the same value from the lower limit of the parameter. This constant value is called α derived from the formula α = (F)^1/4^, where F is the number of factorial points. The range of parameters and the results of the experiments are given in Table 2. Experimental results and predicted results were obtained from laboratory studies and model, respectively.

**Table 2:** CCD of independent variables with their corresponding experimental and predicted recoveries percent.

Variables	Unit	Symbols	Level of variables					
			− α	− 1		0	+ 1	+ α
Adsorbent mass	g	A	0.01	0.02		0.03	0.04	0.05
Sonication time	min	B	1	3		5	7	9
pH of solution	–	C	2	4		6	8	10
Eluent volume	µL	D	100	150		200	250	300

Run	Variables				%Recoveries AO		%Recoveries MB	
	A	B	C	D	Observed	Predicted	Observed	Predicted
1	0	2	0	0	88.99	88.24	85.73	85.71
2	0	0	0	0	97.25	97.00	93.81	93.71
3	0	0	0	2	85.95	85.50	88.12	87.97
4	1	1	1	− 1	73.38	74.01	74.23	74.25
5	− 1	1	1	− 1	85.59	85.92	83.73	83.78
6	0	0	0	0	96.93	97.00	94.10	93.71
7	0	0	2	0	95.41	94.46	90.53	90.34
8	1	1	− 1	1	82.95	83.00	87.38	87.45
9	1	− 1	− 1	− 1	75.59	76.22	77.02	77.27
10	− 1	− 1	− 1	− 1	84.92	84.95	85.73	85.67
11	0	0	0	0	96.28	97.00	93.34	93.71
12	2	0	0	0	84.76	83.75	85.04	84.75
13	− 1	− 1	− 1	1	86.21	86.45	87.17	87.26
14	− 1	1	− 1	1	82.71	83.13	83.81	83.78
15	− 2	0	0	0	93.97	93.60	93.24	93.20
16	− 1	− 1	1	1	97.07	97.00	96.61	96.88
17	0	− 2	0	0	92.11	91.47	93.83	93.52
18	0	0	0	− 2	63.18	62.25	68.03	67.86
19	1	− 1	1	1	97.32	98.06	94.31	94.25
20	1	− 1	1	− 1	73.82	73.92	78.33	78.58
21	0	0	− 2	0	83.45	83.01	80.11	79.98
22	1	1	1	1	95.27	95.76	92.51	92.78
23	0	0	0	0	96.69	97.00	93.54	93.71
24	0	0	0	0	97.69	97.00	93.87	93.71
25	− 1	1	− 1	− 1	83.89	84.02	79.16	79.33
26	1	1	− 1	− 1	72.52	73.11	73.58	73.52
27	1	− 1	− 1	1	88.33	88.51	88.17	88.33
28	− 1	1	1	1	96.63	96.87	92.96	92.82
29	0	0	0	0	97.17	97.00	93.60	93.71
30	− 1	− 1	1	− 1	82.83	83.65	90.65	90.70

The ANOVA is performed to investigate the effect of each variable on the response and also the fitness of the obtained equation with the experimental results. Thus, the p-value at the 95% confidence level is 0.05. If the calculated p-value for each factor is less than 0.05, the factor is significant. On the other hand, if it is more than 0.05, changing that factor has no significant effect on the response^37,38^. Moreover, a lack-of-fit with a *p*-value > 0.05 indicates that the model error is not significant, and the residual is due to a random error. The *p*-values and parameter coefficients for the AO and MB dyes are given in Table 3. As shown in Table 3, the *p*-values of the proposed models are less than 0.05, and the *p*-values of the lack-of-fit are greater than 0.05. Therefore, there is a good agreement between the model and experimental results. In addition, the correlation coefficients can also be used to evaluate the model’s validity. The values of coefficient of determination (R^2^) and adjusted coefficient of determination (Adj-R^2^) are shown in Table 3. The closer R^2^ is to 1, the more variability the model explains and the better it can predict the response. Also, the higher values of Adj-R^2^ and its closeness to R^2^ determine the validity of the proposed model. The R^2^ values of AO and MB are 0.9995 and 0.9994, respectively, suggesting a reasonable agreement between the experimental results. These values indicate that the model can describe more than 99% of the response changes in terms of variables. In addition, Adj-R^2^ is high enough (Adj-R^2^ = 0.9988 for AO and Adj-R^2^ = 0.9933 for MB) that the model can be considered reliable. The proposed quadratic model for the effective extraction of AO and MB dyes is expressed as Eqs. (5) and (6).

5 $$\begin{aligned} \% {\text{ER}}_{{{\text{AO}}}} = & + 97.00 - 2.46{\text{A}} - 0.80{\text{B}} + 2.86{\text{C}} + 5.81{\text{D}} - 0.54{\text{AB}} - 0.24{\text{AC}} \\ & + 2.69{\text{AD}} + 0.80{\text{BC}} - 0.59{\text{BD}} + 2.96{\text{CD}} \\ & - 2.08{\text{A}}^{2} - 1.78{\text{B}}^{2} - 2.06{\text{C}}^{2} - 5.78{\text{D}}^{2} \\ \end{aligned}$$

6 $$\begin{aligned} \% {\text{ER}}_{{{\text{MB}}}} = & + {93}.{71} - {2}.{\text{11A}} - {1}.{\text{95B}} + {2}.{\text{58C}} + {5}.0{\text{2D}} + 0.{\text{64AB}} - 0.{\text{92AC}} \\ & + {2}.{\text{37AD}} - 0.{\text{14BC}} + 0.{\text{71BD}} + {1}.{\text{15CD}} \\ & - {1}.{\text{18A}}^{{2}} - {1}.0{\text{2B}}^{{2}} - {2}.{\text{13C}}^{{2}} - {3}.{\text{94D}}^{{2}} \\ \end{aligned}$$

**Table 3:** Analysis of variance (ANOVA) of remove AO and MB.

Source	DF	AO				MB			
		Sum of squares	Mean square	F-value	*P*-value	Sum of squares	Mean square	F-value	*P*-value
Model	14	2455.19	175.37	308.47	< 0.0001	1609.82	114.99	1674.64	< 0.0001
A- Adsorbent mass	1	145.48	145.48	255.90	< 0.0001	107.06	107.06	1559.21	< 0.0001
B- Sonication time	1	15.67	15.67	27.56	< 0.0001	91.38	91.38	1330.79	< 0.0001
C- pH of solution	1	196.71	196.71	346.01	< 0.0001	160.94	160.94	2343.92	< 0.0001
D- Eluent volume	1	810.73	810.73	1426.05	< 0.0001	606.72	606.72	8836.07	< 0.0001
AB	1	4.76	4.76	8.38	0.0111	6.72	6.72	97.88	< 0.0001
AC	1	1.00	1.00	1.75	0.2057	13.78	13.78	200.73	< 0.0001
AD	1	116.48	116.48	204.88	< 0.0001	89.92	89.92	1309.54	< 0.0001
BC	1	10.26	10.26	18.04	0.0007	0.33	0.33	4.86	0.0436
BD	1	5.75	5.75	10.11	0.0062	8.17	8.17	118.92	< 0.0001
CD	1	140.36	140.36	246.90	< 0.0001	21.18	21.18	308.50	< 0.0001
A2	1	118.92	118.92	209.17-	< 0.0001	38.40	38.40	559.26	< 0.0001
B2	1	87.49	87.49	153.89	< 0.0001	28.72	28.72	418.24	< 0.0001
C2	1	117.07	117.07	205.92	< 0.0001	125.40	125.40	1826.35	< 0.0001
D2	1	917.04	917.04	1613.05	< 0.0001	427.84	427.84	6230.95	< 0.0001
Residual	15	8.53	0.57			1.03	0.069		
Lack of Fit	10	7.34	0.73	3.09	0.1123	0.66	0.066	0.91	0.5823
Pure Error	5	1.19	0.24			0.37	0.073		
Cor Total	29	2463.71				1610.85			

Model summary statistics									
Precision	AO				MB				
	R2	R2-Adj		R2-Pred	R2		R2-Adj		R2-Pred
	0.9995	0.9933		0.9821	0.9994		0.9988		0.9973

The obtained response equations include principal, interaction, and curvature effects. Positive coefficients indicate that increasing the value of these variables in the defined range increases the extraction efficiency. In contrast, negative coefficients indicate that the extraction efficiency is desirable in smaller quantities of these variables.

**Figure 3:**
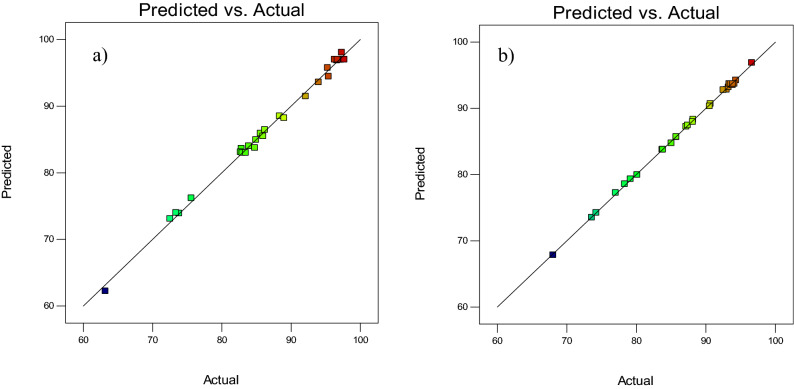
Predicted versus experimental data for extraction of (**a**) AO and (**b**) MB (Image is created by using Design-Expert software version 10).

Comparing the predicted responses of the model and the actual values is another factor in evaluating the model’s validity. This comparison is presented in Fig. 3 in the form of a graph containing the predicted responses of the model and the actual values. The closeness of the obtained points to the 45° line suggests a good agreement between the proposed model and the experimental data, thereby confirming the model’s validity.

**Figure 4:**
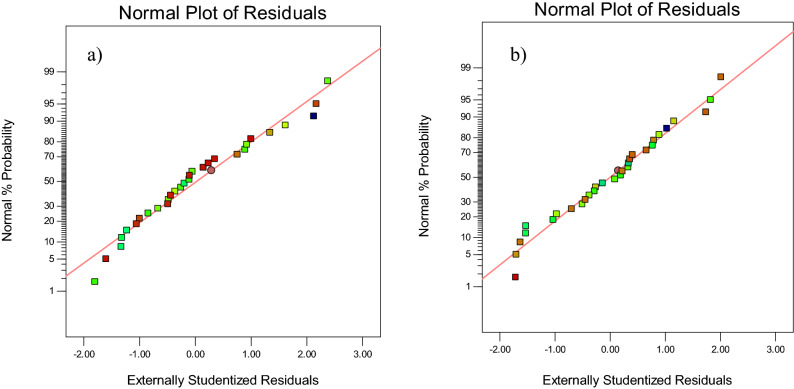
The plots of normal probability of the residuals for extraction of (**a**) AO and (**b**) MB (Image is created by using Design-Expert software version 10).

Figure 4 shows the normal probability of the responses. This plot illustrates the distribution pattern of the errors. The errors are defined as differences between the experimental values and the predicted values of the model responses. Proper and normal distribution of points around the straight line indicates a proper distribution of errors. According to these plots, as the errors are normally distributed, the models are significant, and the predicted responses are consistent with the experimental data.

### Response surface plots

**Figure 5:**
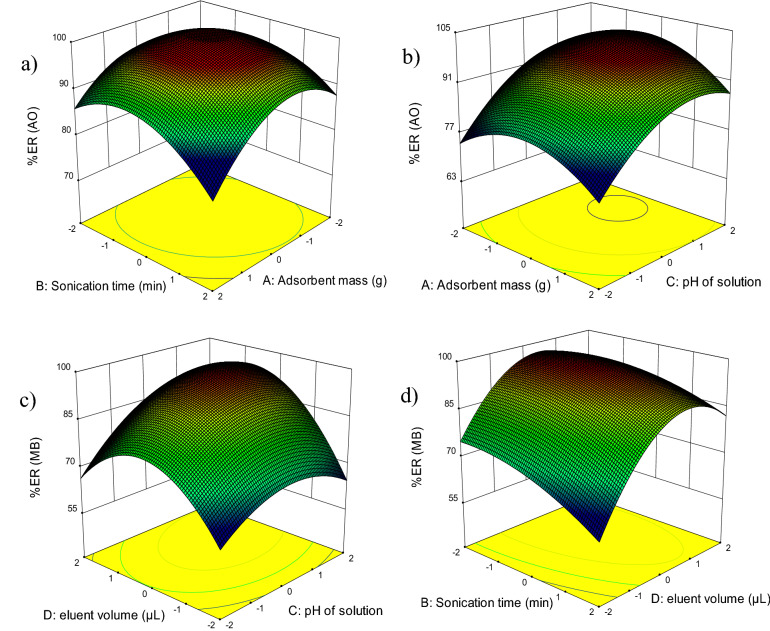
Three-dimensional plots of the interaction effects between variables and extraction efficiency of (**a**), (**b**) AO and (**c**), (**d**) MB (Image is created by using Design-Expert software version 10).

The ultimate objective of designing the experiment and presenting the model is to achieve a condition of the experimental variables under which the system response (peak area of the target analytes) is within the maximum achievable value. Factors affecting the extraction process have interaction effects on the responses and their independent effect. The independent and interaction effects of the studied parameters on the extraction efficiency were studied using three-dimensional plots, including the peak area of the target analytes against two independent parameters. The three-dimensional plot of the response as a function of two variables by keeping other variables constant at fixed levels (central level) leads to a better understanding of these two variables’ effects and interactions and shows the optimum reaction conditions. Figure 5a–d demonstrate interactions between the independent variables and the desired response. Optimum conditions can also be attained from these plots.

As shown in Fig. 5a, the AO dye extraction efficiency increased with increasing extraction time and the adsorbent amount. According to the figure, the extraction rate increased with increasing the adsorbent amount. As the mass of adsorbent increases, more sites will be available; thus, the dye adsorption on the adsorbent surface would increase. The optimum value for the adsorbent amount was 0.025 g, as exceeding the adsorbent amount higher than 0.025 g did not change the dye extraction percentage so much. Therefore, to minimize the amount of adsorbent consumption, 0.025 g was selected as the optimum amount. Sharifi et al. (2021) have observed similar results in assessing the effect of adsorbent amount on the extraction of crystal violet (CV) and auramine O (AO) dyes. In this study, nano-mesoporous MCM-41 @ SiO2-NH-pydc was used as the adsorbent. According to the results, increasing the adsorbent amount increased the extraction of CV and AO dyes^39^. In another research, Pataer et al. (2019) used molecularly imprinted polymer to extract auramine O (AO) dye. The results showed that increasing the adsorbent amount increased the extraction efficiency, which is consistent with the present study results^40^.

Figure 5b illustrates the simultaneous effect of pH and the adsorbent amount on the AO dye extraction efficiency. As can be seen, pH has a more significant effect on extraction efficiency than the adsorbent amount, indicating that increasing the pH leads to a further increase in the peak areas. The pH of the solution is one of the most important parameters affecting the extraction process. The effect of pH was examined in the range of 2–10. As shown in Fig. 5b, the extraction efficiency increases with increasing pH. The pH_pzc_ of MgO is 4.4^41^. In pH $<$ pH_pzc_, there is a positive charge on the adsorbent surface that creates a repulsion between the positive surface of the adsorbent and the positively charged cationic dyes. However, at pH $>$ pH_pzc_, the electrostatic repulsion between the dye and the adsorbent surface decreases, increasing the dye extraction. The highest amount of dye extraction was obtained at pH = 7. The results of the present study are consistent with those of Hakami et al. (2021), and Sha et al. (2021)^42,43^.

The eluent volume is another parameter affecting the extraction process. In this study, different volumes of the optimal solvent were investigated for dye extraction, and the optimal volume of eluent solvent (acetone) for both analytes was selected to be 225 μL. The results in Fig. 5c show that in more than 225 μL, all the dye enters the eluent, and the equilibrium moves quantitatively toward the eluent and becomes completely desorbed. Zhang et al. (2021) obtained similar results in the extraction of methylene blue (MB) dye using alumina-neutral (ALN) cartridges^44^. Dil et al. (2016) extract safranin O dye using activated carbon modified with Fe_2_O_3_ nanoparticles (Fe_2_O_3_-NPs-AC) as an adsorbent. The effects of various factors like adsorbent amount, eluent volume, ultrasound time, and solution pH were assessed in dye extraction. Consistent with the present study results, the results showed that increasing eluent volume increased the dye extraction rate^45^.

Extraction time was examined in the range of 1–9 min. As shown in Fig. 5d, with increasing ultrasound time, there is more time to expose dye and adsorbent molecules. Certainly, the greater amount of dye is absorbed by the adsorbent in the initial moments, and the increase in time is to complete the adsorption process. The optimum time for this process was 5 min. These results are consistent with the study of Asfaram et al. (2016), who investigated simultaneous extraction of auramine O (AO) and malachite green (MG) dyes by ultrasound from aqueous solutions. Asfaram et al. utilized Mn-doped ZnS nanoparticles loaded on activated carbon as adsorbents. The variables and the designed experiments were examined using the RSM. Adsorbent amount (1.2 mg), ultrasound time (3.7 min), 150 μL eluent, and pH = 8 were considered optimal conditions. This study showed that increasing ultrasound time increased the AO and MG dye extraction^46^.

### Analytical figures of merit

The potential of the UA-DMNSPME-UV/Vis method in AO and MB dyes extraction was evaluated by investigating the figures of merit of the method under optimum conditions. Based on the obtained results (Table 4), the linear dynamic range (LDR) of AO and MB was in the range of 1–1000 ng mL^−1^ and 5–2000 ng mL^−1^, respectively. Also, the R^2^ for the obtained linear ranges was greater than 0.9985, and the LODs for the AO and the MB dyes were 0.33 ng mL^−1^ and 1.66 ng mL^−1^, respectively. The extraction recovery percentage (ER%) and PF of the method were determined using Eqs. (3) and (4), respectively. Based on the obtained results, the method efficiency for AO and MB dyes was 92.85%-99.57%, and the PF was 44.5. The method’s reproducibility was shown by the relative standard deviation (RSD). In this study, 5 repeated extractions were performed to determine RSD in each analyte measurement. Also, measurements of solutions with a concentration of 500 ng mL^−1^ were performed, and the peak areas were analyzed. The results show that the RSD of these measurements is less than 3.1.

**Table 4:** Analysis performance of AO and MB extracted with the MgO-NPs.

Analytes	LDR^a^	Regression equation	Correlation coefficient	LOD^b^	RSD^c^	PF^d^	Recoveries (%)
AO	1–1000	y = 0.0013x- 0.0059	0.9986	0.33	3.1	44.5	94.73–99.57
MB	5–2000	y = 0.0007x- 0.0092	0.9985	1.66	2.9	44.5	92.85–99.36

^a^Linear dynamic range (ng mL^−1^), ^b^Limit of detections (ng mL^−1^), ^c^Relative standard deviation (n = 5), ^d^Preconcentration factor.

### Optimization of process

The desirability function is the most important and common method used in the simultaneous optimization of analytical processes^47,48^. In this function, 0 denotes a completely undesirable response, and 1 is for a perfectly desirable response. The desirability function is an effective and cost-effective method in multi-response optimization in analytical chemistry. Design-Expert software was used to determine the RSM method’s optimum levels. Based on the results and as the highest percentage of extraction was desirable, the optimum conditions determined by the software include the pH solution of 7, the adsorbent amount of 0.025 g, sonication time of 5 min, and eluent volume of 225 μL. The extraction percentage of AO and MB dye in the proposed optimum points was 98.89 ± 2.3% and 96.92 ± 2.8% (n = 5), respectively.

### Interference studies

We investigated the effect of the matrix on the selectivity of the AO and MB extraction process using MgO-NPs, as an adsorbent in competition with other ions present in the solution. In this study, the acceptable concentration causing a change in extraction was considered ± 5%. The effect of these counterions is given in Table 5. As can be seen in the extraction results, most of the studied species do not show interference and negative effect even at high concentrations, suggesting the selectivity of the AO and MB dye extraction process using this adsorbent.

**Table 5:** Effects of influence substances on the determination of AO and MB.

Foreign species	Spiked concentration	%ER	
	(μg mL^−1^)	AO	MB
Li^+^, Na^+^, K^+^ Mg2^+^, Ca2^+^, F^−^, C^−^, Br^−^	100	97.41	98.53
Co^2+^, NO^3−^, Fe^2+^, Fe^3+^, Pb^2+^	70	99.56	98.85
Al^3+^, Cd^2+^, Ni^2+^	50	98.37	97.20
Ag^+^, Cu^2+^, Sn^2+^	10	99.12	98.94

### Reusability of the MgO-NPs

**Figure 6:**
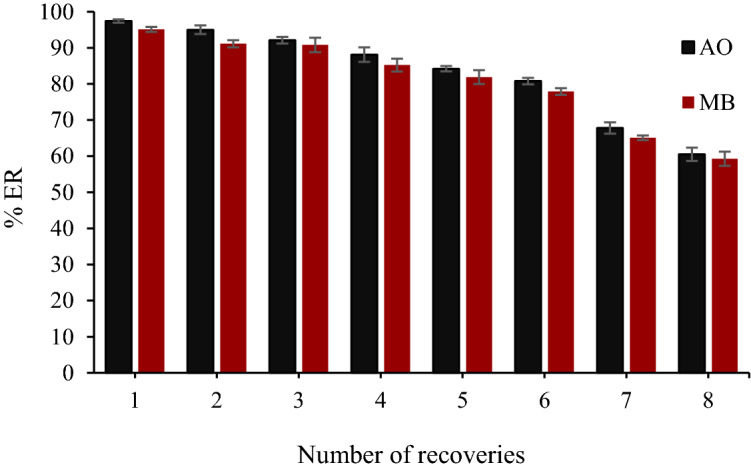
Regeneration studies of magnetic MgO-NPs (Sample volume: 10 mL, eluent volume: 225 µL, adsorbent mass: 0.025 g, sonication time: 5 min, and pH: 7).

In order to show the stability of the adsorbent, magnetic MgO-NPs were used several times under optimal conditions. After each extraction test, the adsorbent was removed by applying an external magnet and washed with acetone. After six runs, the extraction rate was about 80% (Fig. 6). This reduction in extraction rate is probably due to (1) the partial degradation of the adsorbent structure in the process of chemical regeneration, (2) the presence of dye impurities, and (3) the occupation of some of the active adsorbent sites^49^. The results of reusability studies show that the adsorbent has good recovery ability and is a suitable candidate for industrial applications as an adsorbent.

### Real samples analysis

Ambient water samples were used to evaluate the efficiency of the proposed method in determining AO and MB dyes. The real samples used to determine AO and MB dyes by UA-DMNSPME-UV/Vis using MgO-NPs were: tap water, wastewater, fish farm, and lake water. The suspended particles were removed by passing these water samples through a filter paper. Due to the absence of dye in real samples, different dye concentrations were added to the samples. Then, the added value of each was determined by standard method and after DMNSPME by UV/Vis spectrophotometer. The results are presented in Table 6. These results indicate that the change in the matrix of the samples has no significant effect on the extraction of target analytes of the samples. Relative yields calculated using Eq. (4) are in the range of 91.22–99.12%.

**Table 6:** Determination of AO and MB in the environmental water samples.

Samples	Analyte	Add (ng mL^−1^)	Found (ng mL^−1^)	%ER ± %RSD (n = 3)
Tap water	AO	50	49.24	98.48 ± 2.8
		100	98.45	98.45 ± 3.3
		500	495.60	99.12 ± 2.1
	MB	50	48.72	97.44 ± 2.8
		100	97.39	97.39 ± 3.4
		500	489.84	97.96 ± 3.7
Wastewater	AO	50	47.08	94.16 ± 2.5
		100	95.93	95.93 ± 2.2
		500	469.57	93.91 ± 3.6
	MB	50	48.93	97.86 ± 2.7
		100	98.69	98.69 ± 2.6
		500	476.14	95.22 ± 1.8
Fish farm	AO	50	46.98	93.96 ± 2.1
		100	96.23	96.23 ± 3.3
		500	472.69	94.53 ± 2.4
	MB	50	48.97	97.94 ± 1.7
		100	98.06	98.06 ± 2.3
		500	482.52	96.50 ± 2.9
Lake water	AO	50	47.55	95.10 ± 3.2
		100	96.37	96.37 ± 2.4
		500	488.40	97.68 ± 2.8
	MB	50	45.61	91.22 ± 3.1
		100	98.85	98.85 ± 2.9
		500	470.76	94.15 ± 2.6

### Comparison with other methods

Table 7 presents the comparison results of the UA-DMNSPME-UV/Vis method with other methods for the determination AO and MB dyes. The table also lists the significant parameters of these methods. A point that can be deduced from this table and its comparison with previous results is that a significant amount of dyes are extracted in a very short time (5 min) in the ultrasonic-assisted extraction method. Also, RSD less than 3.1 indicates the high precision of this method compared to other methods in the literature. Short reaction time, high performance, a low number of experiments, the ability to use different types of solvents, low solvent consumption, recoverability of the adsorbent, simplicity, and relatively low cost of preconcentration and determination of AO and MB dyes are the main advantages of this method. As can be seen from Table 7, the UA-DMNSPME-UV/Vis method is easier, faster, and more convenient than other methods. Also, it has high sensitivity and precision than the other techniques. Furthermore, it has a high LOD and PF and reduces environmental issues because of its low solvent consumption.

**Table 7:** Comparison of the present method with other extraction methods for the determination of AO and MB.

Analytical method	Analyte	LDR^a^	LOD^b^	RSD^c^	Application	PF^d^	References
DLLME^e^-UV/Vis	AO	10–2000 ng mL^−1^	2.62 ng mL^−1^	3.2%	Rain water, Tap water, Double-distilled water, Lake water, River water and Wastewater	66.67	^50^
UADSPME^f^	AO	0.4‐9 mg L^−1^	0.0015 mg L^−1^	3.44%	Yasouj city water, Sheshpir minral water and Beshar river water		^51^
DSPE^g^-UV/Vis	AO	5–200 mg L^−1^	1 mg L^−1^	2.4–3.8%	Lake water		^52^
AA-IL-DLLME^h^-HPLC	AO	0.05–50 μg g^−1^	0.01 μg g^−1^	2.7–7.4%	Tofu, Dried bean curd, Dried beancurd stick and Bean curd skin		^53^
SPE-HPLC	AO	0.01–40 mg kg^−1^	0.003 mg kg^−1^	3.2–5.5%	Soybean products, Soybean products and Yellow croaker		^54^
DSPME-UV/Vis	AO	1–2000 ng mL^−1^	0.23 ng mL^−1^	2.2%	Rain water, Tap water, Double-distilled water, Mineral water and Wastewater	100	^55^
SPE-HPLC	AO	50–100,000 ng mL^−1^	1.25 ng mL^−1^	3.7–7.7%	curry paste, chili sauce, gochujang, tandoori chicken, shrimp powder, and powder soup		^56^
UA-DMNSPME^i^-UV/Vis	AO	1–1000 ng mL^−1^	0.33 ng mL^−1^	3.1%	Tap water, Wastewater, and Lake water	44.5	This work
LLE^j^-CE	MB	1000–60,000 ng mL^−1^	1000	7.1%	Human urine	4.7	^57^
DSPE–CPE	MB	2–350 μg L–1	0.65 μg L^−1^	1.05%	Domestic wastewater, Beshar water and Karoon water	100	^58^
HF-LPME^k^-HPLC	MB	1.6–600 ng mL^−1^	0.5 ng mL^−1^	3.8%	River water, Sea water and Wastewater	160	^59^
SALLE^l^-UV/Vis	MB	200–7000 ng mL^−1^	60 ng mL^−1^	1.1–3.8%	Wastewater	-	^60^
SALLME-BE^m^-UV/Vis	MB	2–170 ng mL^−1^	0.5 ng mL^−1^	3.3–6.2%	River water and Wastewater		^61^
UA-DMNSPME-UV/Vis	MB	5–2000 ng mL^−1^	1.66 ng mL^−1^	2.9%	Tap water, Wastewater, Fish farm, and Lake water	44.5	This work

^a^Linear dynamic range, ^b^Limit of detection, ^c^Relative standard deviation, ^d^Preconcentration factor, ^e^Dispersive liquid–liquid microextraction, ^f^Ultrasound-assisted dispersive solid-phase microextraction, ^g^Dispersive solid-phase extraction, ^h^Air-assisted ionic liquid-based dispersive liquid–liquid microextraction, ^i^Ultrasound-assisted dispersive-magnetic nanocomposites-solid-phase microextraction, ^j^Liquid-liquid extraction, ^k^Hollow fiber liquid-phase microextraction, ^l^Salting-out assisted liquid–liquid extraction, ^m^Shaker-assisted liquid–liquid microextraction combined with back-extraction.

## Conclusion

This study used the UA-DMNSPME-UV/Vis method to preconcentrate and determine trace amounts of AO and MB dyes from ambient water samples. This research offers a selective, low-cost, and simple method to determine the amount of AO and MB dyes as a dye and aromatic indexes in contaminated wastewaters. In recent years, the development of solid-phase extraction methods has introduced adsorption with appropriate efficiency as a fundamental necessity. Therefore, in this work, MgO-NPs were used as a suitable adsorbent to increase the extraction efficiency. RSM design method was also used to achieve the best optimum results. Indeed, RSM was used to obtain the optimum conditions of process parameters such as solution pH, adsorbent dosage, eluent volume, and ultrasonic time. In this method, pH = 7, eluent volume of 225 μL, the adsorbent dosage of 0.025 g, and time of 5 min were considered the optimum conditions to obtain the maximum extraction of AO and MB dyes. The recovery percentage of AO and MB extraction under optimum conditions for real samples was in the range of 91.22–99.12%. This method has good reproducibility and a wide linear range of 1–1000 ng mL^−1^ for AO and 5–2000 ng mL^−1^ for MB. The LODs for AO and MB were 0.33 ng mL^−1^ and 11.66 ng mL^−1^, respectively. In addition, the adsorbents’ reusability results showed that they could be reused up to 6 times without a significant loss in the percentage of dye extraction. Furthermore, the results of interference studies revealed that the presence of different ions did not significantly interfere with the extraction of AO and MB. Hence, the UA-DMNSPME-UV/Vis method can be proposed as an efficient method for extracting desired dyes from water and wastewater samples.

## Data Availability

All data generated or analysed during this study are included in this published article.
